# Design and Validation of a Ten-Port Waveguide Reflectometer Sensor: Application to Efficiency Measurement and Optimization of Microwave-Heating Ovens

**DOI:** 10.3390/s8127833

**Published:** 2008-12-03

**Authors:** Juan L. Pedreño-Molina, Juan Monzó-Cabrera, Antonio Lozano-Guerrero, Ana Toledo-Moreo

**Affiliations:** 1 Departamento de Tecnologías de la Información y Comunicaciones, Universidad Politécnica de Cartagena / Campus Muralla del Mar s/n, 30202 Cartagena, Murcia, Spain; E-Mails: Juan.Monzo@upct.es (J.M.); Antonio.Lozano@upct.es (A.L.); 2 Departamento de Tecnología Electrónica, Universidad Politécnica de Cartagena / Campus Muralla del Mar s/n, 30202 Cartagena, Murcia, Spain; E-mail: Ana.Toledo@upct.es (A.T.)

**Keywords:** Reflectometer, microwave sensor, scattering parameter, neural calibration

## Abstract

This work presents the design, manufacturing process, calibration and validation of a new microwave ten-port waveguide reflectometer based on the use of neural networks. This low-cost novel device solves some of the shortcomings of previous reflectometers such as non-linear behavior of power sensors, noise presence and the complexity of the calibration procedure, which is often based on complex mathematical equations. These problems, which imply the reduction of the reflection coefficient measurement accuracy, have been overcome by using a higher number of probes than usual six-port configurations and by means of the use of Radial Basis Function (RBF) neural networks in order to reduce the influence of noise and non-linear processes over the measurements. Additionally, this sensor can be reconfigured whenever some of the eight coaxial power detectors fail, still providing accurate values in real time. The ten-port performance has been compared against a high-cost measurement instrument such as a vector network analyzer and applied to the measurement and optimization of energy efficiency of microwave ovens, with good results.

## 1. Introduction

Microwave-heating applications are slowly increasing their importance due to the recent applications to the food industry, sanitary sector, chemical and pharmaceutical engineering and polymer production, among others. Although this technology is mature and can offer several advantages such as reduction of processing times, usage of clean energy and the resulting reduction of atmospheric pollution, it has to compete against cheaper energies based on combustion to generate heat. Therefore, one of the main goals of microwave heating technology at industrial applications is the monitoring of energy efficiency for the optimization and detections of malfunctions of the system.

The power efficiency of a microwave oven can be easily related to the reflection coefficient at the feeding port. The conventional non-invasive measurement techniques for the reflection coefficient are often based on directional couplers that separate incident and reflected power within the waveguide. The comparison of both contributions allows the estimation of the magnitude and phase of the reflection coefficient. To measure the reflection coefficient, Vector Network Analyzers (VNAs) and Six-Port Reflectometers (SPRs) are by far the most widely used instruments. Calibration is an essential step to guarantee accurate measurements with such instruments, since noise, the phase error introduced by the cables and non-linear behavior of detectors may lead to high error levels.

VNAs are very high precision instruments that can be used at laboratory stages but, due to their high price, they are very seldom used at industrial sites. Additionally, the VNA configuration does not allow handling high-power levels easily. Therefore, SPRs are often used and are the preferred sensors for monitoring the reflection coefficient both at high and low power levels. This SPR is particularly interesting thanks to the use of power detectors instead of mixers and directional couplers, thus providing simpler circuits when compared to VNA configurations.

Several techniques for calibration of SPRs have been previously published [1-3]. These studies consider aspects such as dynamic range and non-linearity of power measuring diodes [1, 2]. The most extended calibration technique is based on the use of four standard loads whose reflection coefficients are very precisely known [3]. With this technique, the numerical solution for calibration equations can be represented in the complex plane by means of three circles. The intersection of the circles provides the desired solution for the calibration process.

Due to inherent noise and other measurement errors, the intersection point of the circles is extended in practice to a less precise area and error minimization techniques must be used in order to reduce the influence of these errors. A new calibration method based on Fourier coefficients proximity for SPR parameters was presented in [4] in order to reduce the calibration uncertainty. A calibration method based on the use of phase shifters and attenuators was also proposed in [5]. In [6], the calibration method is based on the analytical description of the communication system behavior and the measurements performed when two signals with a slight frequency difference are connected to the reflectometer's inputs.

Several calibration techniques propose the characterization of the diode behavior in order to improve calibration and measurement performance [7, 8]. Some examples of linear approximations for diode response versus frequency and operation temperature can be found at [9, 10]. Other alternatives that use thermistors for temperature control and monitoring are described in [11].

Calibration methods based on artificial neural networks (ANNs) were proposed in [12, 13]. This approach is advantageous for permitting automatic calibration procedures, although ANNs require a large number of known standards for training the network. However, automatic reflection coefficient generators can be easily built. In fact, some recent studies have shown that sample movement within microwave ovens may generate large variations for reflection coefficient at the feeding port [14] and that they can be used as low-cost impedance generators.

In this work, a new ten-port waveguide reflectometer based on low-cost power detectors is presented. Eight coaxial probes are inserted within the waveguide in order to sample the standing wave present at the waveguide and thus to estimate reflection coefficient. The device is analyzed with CST Microwave Studio electromagnetic (EM) commercial software in order to ensure monomode working conditions. The electronic design of the built power measuring circuits is shown. The calibration procedure, based in ANN learning techniques, is also described. The ten-port sensor has been built and validated by obtaining both magnitude and phase values of reflection coefficient and comparing them to VNA performance, using the Industrial, Scientific and Medical (ISM) 2.45 GHz frequency for demonstration purposes. Finally, as an example of application, this sensor has been used to measure and optimize the energy efficiency of a microwave oven. The optimization process is based on placing the sample at the optimal position within the microwave cavity. An example of this procedure is described in this paper.

## 2. Basic Theory and design principles for a ten-port reflectometer

### 2.1. Six-port reflectometer review

A six-port reflectometer consists of a simple circuit with two ports for signal input and output, and four ports with their corresponding power detectors that sample the standing wave within the transmission line. The input port is connected to the EM source and the output port is connected to the load. This load can generate a mismatch that leads to reflections whose magnitude must be measured by the reflectometer.

**Figure 1.**
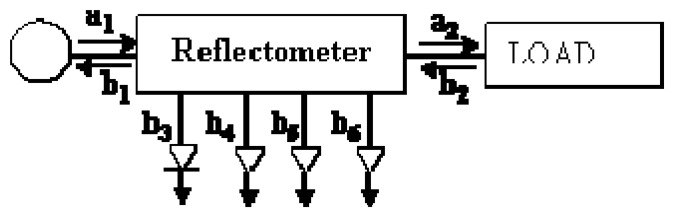
Six-port reflectometer scheme.

Figure 1 shows a simplified scheme of a six-port reflectometer, where *a_i_* signals go always into the *i^th^* port and *b_i_* signals go out from the *i^th^* port. Ports ranging from 3 to 6 are matched and therefore no reflected wave is considered there. In this scheme, Port 1 is the input port where the EM source is connected, whereas Port 2 is the output port of the sensor.

The complex reflection coefficient (*Γ*), also called *S_11_* scattering parameter, provides a relationship between the incident wave amplitude at Port 1 (*a_1_*) and the reflected wave amplitude (*b_1_*) at the input port (Port 1 in Figure 1). The bigger the magnitude of *Γ*, the more energy is reflected to the EM source, thus less energy is absorbed by the load.

A numerical relationship can be obtained from the power detected at Ports 3 to 6 to determine the reflection coefficient in the load. This numerical expression combines the value of nine complex parameters, very sensitive to measurement noise and non-linearities, in order to determine the desired reflection coefficient value.

The equation system shown in (1) relates the incident and reflected wave amplitudes at Port 2 to the sampling amplitudes. The calibration procedure of this six-port reflectometer is based on finding the numerical solution for this equation system, where *M_i_* and *N_i_* are complex constants. As described earlier, several calibration methods and load standards can be used.

(1) $$\begin{array}{ll} b_{3} & =M_{3}\cdot a_{2}+N_{3}\cdot b_{2}\\ b_{4} & =M_{4}\cdot a_{2}+N_{4}\cdot b_{2}\\ b_{5} & =M_{5}\cdot a_{2}+N_{5}\cdot b_{2}\\ b_{6} & =M_{6}\cdot a_{2}+N_{6}\cdot b_{2} \end{array}\}$$

To solve this system, eight linear equations must be considered. A theoretical linear dependence of all important parameters could be obtained if noise, measurement perturbations and non-linear effects are not considered. However, the calibration procedure becomes a hard task under real conditions where the above mentioned effects can not be neglected.

Additionally, this configuration cannot be used when any of the power detectors is broken, damaged or saturated. This is due to the fact that the six-port uses the minimum amount of detecting ports that are required to find a precise solution. This may lead to delays both in laboratory measurements or industrial monitoring since the power detector must be repaired or changed, and then the whole device recalibrated, before being able to measure again.

### 2.2. Ten-port description

**Figure 2.**
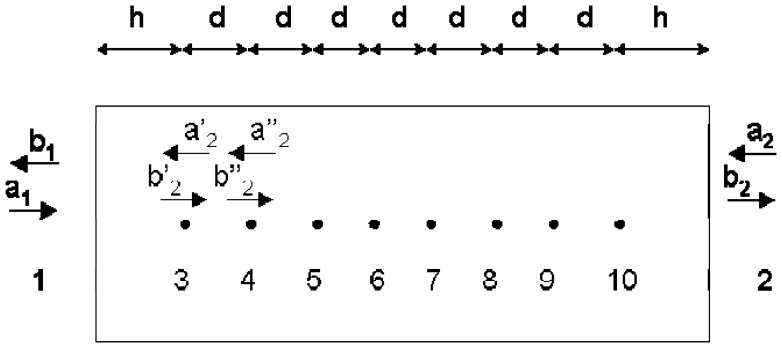
Ten port scheme with input and output ports and sampling coaxial ports, *d* being the distance between consecutive coaxial probes within the waveguide.

In this work a new ten-port reflectometer configuration is presented. In this case, a standard WR-340 waveguide section (4.3 cm × 8.6 cm) has been used. Eight equally spaced coaxial probes have been inserted at the center of the wide wall of the waveguide. These coaxial probes sample the standing wave within the waveguide and therefore provide an estimation of the reflection coefficient. The output of these coaxial probes is connected to a non-linear low-cost power meter. Figure 2 shows the scheme of the proposed configuration where Ports 1 and 2 are respectively connected to the power source and load. Ports ranging from 3 to 10 correspond to the ports of coaxial probes.

The main advantage of this measurement configuration is that only a very small part of the delivered power is absorbed by the sampling probes, which ensures that almost all the delivered power arrives to the sample. This is important mainly for high power applications such as microwave ovens, radar and etc. This structure is therefore different from that used by conventional laboratory equipment such as VNAs.

**Figure 3.**
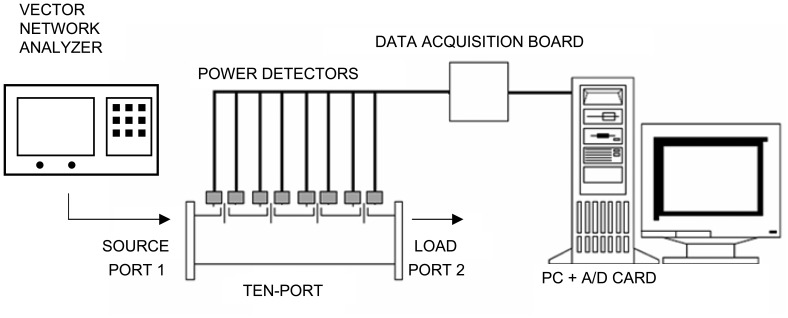
Experimental set up.

Figure 3 shows the measurement system diagram, where the power meters convert the radiofrequency power collected at the coaxial probes into a DC voltage. A personal computer and a data acquisition board are used in order to collect those voltages in real time. Additionally, a VNA is used at Port 1 in order to both generate the incident microwave power and to measure the reference value of the reflection coefficient for calibration purposes. A variable load is employed at Port 2 in order to generate changing values for the reflection coefficient at Port 1. Both the VNA reference measurements and the voltages at each coaxial probe are stored and subsequently processed by a RBF neural network in order to accomplish the calibration process. Contrary to previous work, no linearization process for the power meters is carried out, since this procedure is supposed to be inherently provided by the neural network calibration process.

The measurement procedure is accomplished as follows:

1) The microwave source provides the incident wave (*a_1_*) to the port.
2) The electromagnetic energy propagates along the waveguide reflectometer until it reaches the sample.
3) A reflected wave (*b_2_*) is generated at the variable load thus generating a standing wave within the reflectometer.
4) The power sensors sample the energy of this microwave standing wave and convert the detected power into voltage in a logarithmic way.
5) Simultaneously, a VNA measures the reference value for the reflection coefficient.
6) These voltages obtained from power detectors and the reference reflection coefficient value are then introduced to a neural network. This neural network learns the relationship between the reference value of the reflection coefficient (output of the network) and the output voltages from power detectors (fed as inputs to the neural net).

### 2.3. Simulation of ten-port performance and design principles

**Figure 4.**
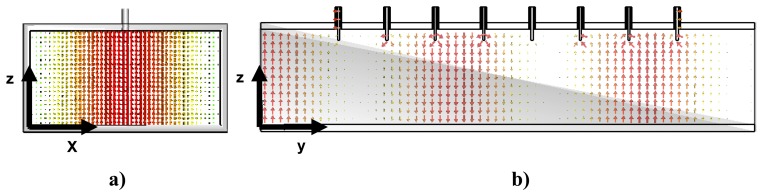
Electric field distribution for TE*_10_* mode at the waveguide. (a) Perpendicular cross section. (b) Along the propagating direction.

The commercial CST Microwave Studio electromagnetic software has been used in order to test the performance of the ten-port reflectometer and to ensure that a monomode behavior is observed at Port 1, where the reflection coefficient is to be measured. In this case it is required that only the TE*_10_* mode, which is the first propagating or fundamental mode of the rectangular waveguide, propagates along the ten-port whose rectangular cross section is *a*=8.6 cm and *b*=4.3 and the working frequency is 2.45 GHz. Figure 4 shows the electric field distribution of the TE*_10_* mode. Figure 4a) shows the electric field distribution at the cross-section perpendicular to the propagation direction and Figure 4b) the distribution for this propagation direction. It can be observed that the maximum value for the electric field is located at the center of the waveguide and that the polarization of the electric field is oriented in the same way that the coaxial probes. Therefore, a good coupling is obtained between the sampling coaxial probes and the electric field.

**Figure 5.**
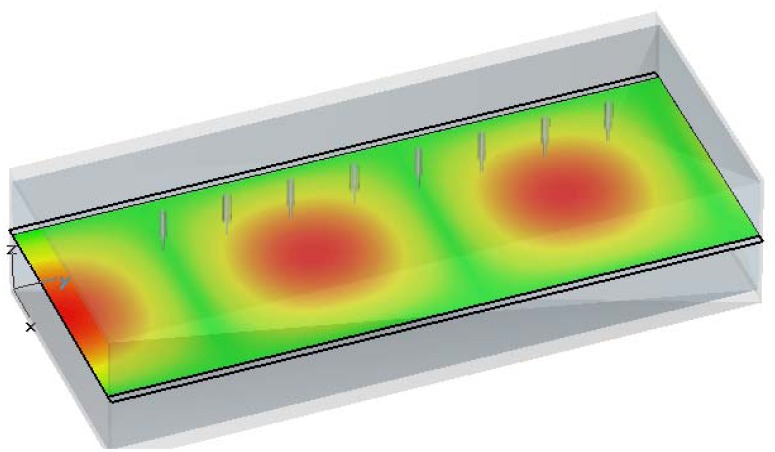
Electric field magnitude across a ten-port, including the effect of the metallic probes.

Figure 5 shows the absolute value of the electric field along the waveguide. Although only one plane is shown, it should be born in mind that the electric field keeps constant along *z* direction, as shown in Figure 4.a). It can be observed that the introduction of metallic probes within the waveguide does not change the TE*_10_* mode spatial distribution.

**Figure 6.**
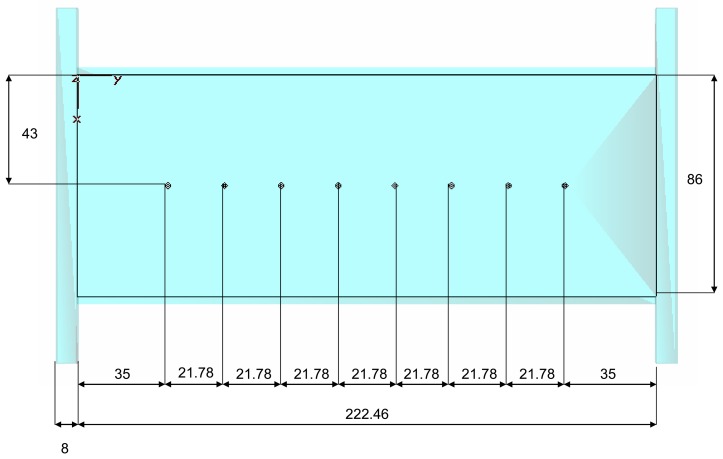
Ten-port length and distances (in mm) between probes.

**Figure 7.**
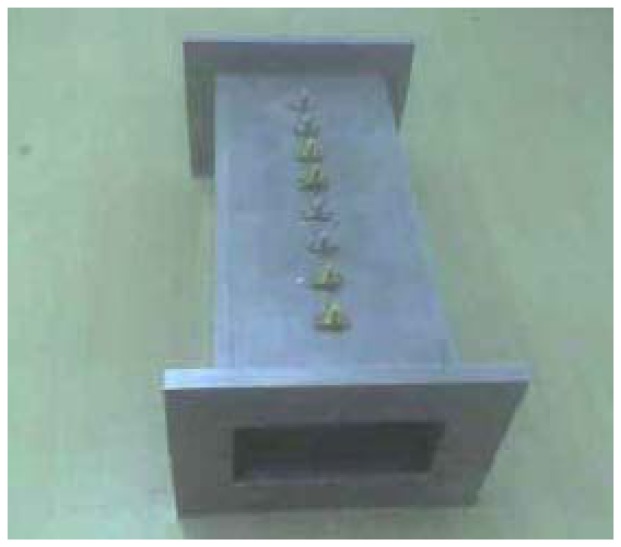
Real implementation of the ten-port reflectometer.

In the WR-340 waveguide, the wavelength is 174.28 mm. Therefore, the ten-port length was fixed at 222.46 mm in order to contain at least this wavelength for the fundamental mode. Figure 6 shows the ten-port length and distances between probes. In Figure 7, a prototype of the real implementation of the ten-port sensor in aluminum can be seen.

### 2.4. Power sensors and microstrip board circuits

Eight logarithmic power sensors have been employed for detecting both the magnitude and phase of the reflection coefficient. Each of the power detectors was associated to each of the coaxial ports. This number doubles the detectors employed in the six-port configuration in order to increase the amount of input data to the neural network and to reduce sampling distance of the standing wave when compared to the SPR configuration. This was supposed to reduce the error during the reflection coefficient estimation. In addition, this also permits the sensor to reconfigure whenever a detector fails, still providing accurate results, as it will be shown later.

Linear Technology LTC5530 (Schottky Diode RF Detector) power detectors were employed at each coaxial port. These detectors are non-linear and their working bandwidth covers from 300 MHz up to 7GHz. The input dynamic range allows power levels from -32 dBm to 10 dBm and the output DC-voltage range operates from 2.7V to 6V. Therefore, these power detectors transform the input radiofrequency power into a DC signal with a non-linear relationship.

**Figure 8.**
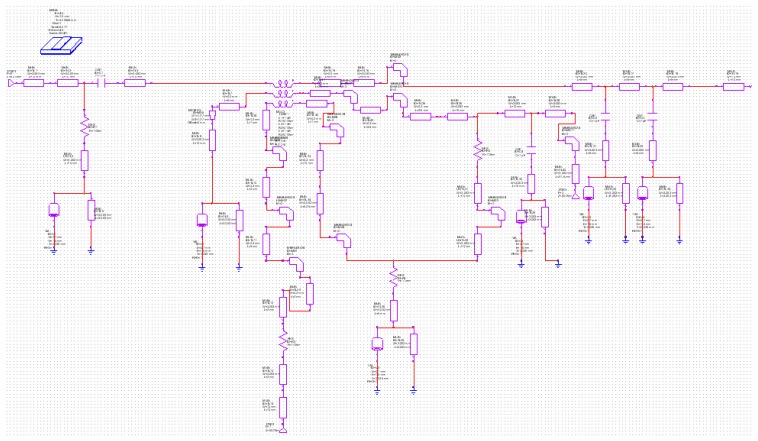
Schematic of the microstrip board for power detector use.

**Figure 9.**
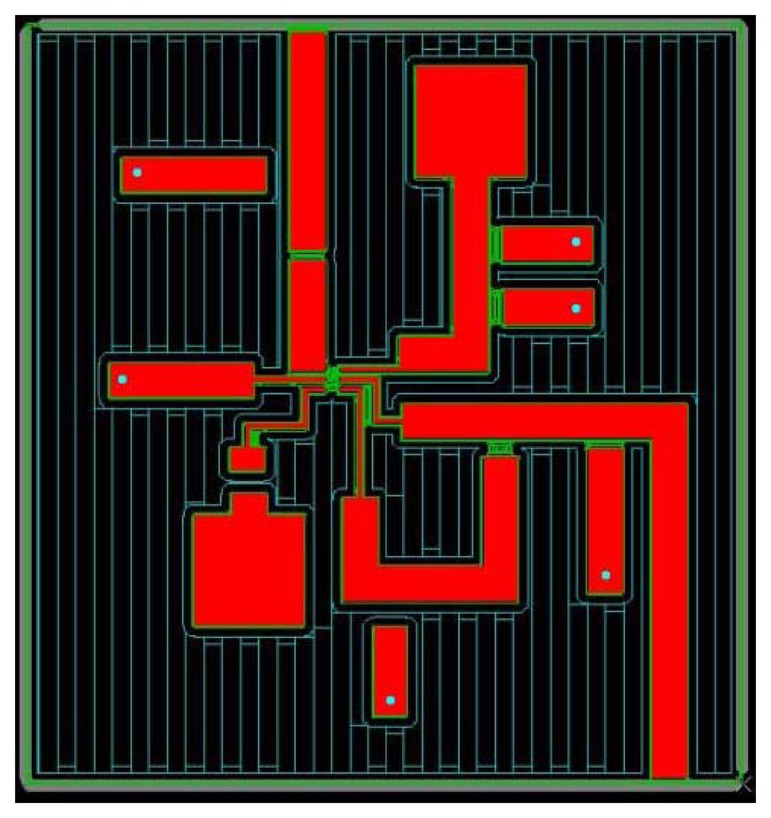
Layout of the microstrip board for power sensors.

It was necessary to include these integrated circuit power detectors within a microstrip board, in order to provide a coaxial to microstrip transition and to make available the necessary DC bias and ground for proper detector working. Commercial Microwave Office software was employed to design the microstrip board. Figure 8 shows the corresponding schematic circuit and Figure 9 its layout. Circuit Cam software was used in order to manufacture the physical circuit.

### 2.5. Neural network for calibration

As described previously, conventional calibration of six-port reflectometers is based on the use of standard loads and several mathematical relationships which deal with complex numbers and that usually require optimization tasks to reduce the influence of measurement noise and power detectors non-linearity on final sensor accuracy. On the contrary, in this work we present a different calibration process based on the usage of neural networks to relate the DC voltage values provided by each power detector to the reference reflection coefficient measurement. Therefore, a ZVRE Rohde & Schwarz VNA was employed during the calibration procedure both for providing the 2.45 GHz incident signal at Port 1 and to measure the reference value of the reflection coefficient also at Port 1. The power level used both in calibration procedures and measurements was set to 0.5 watts.

**Figure 10.**
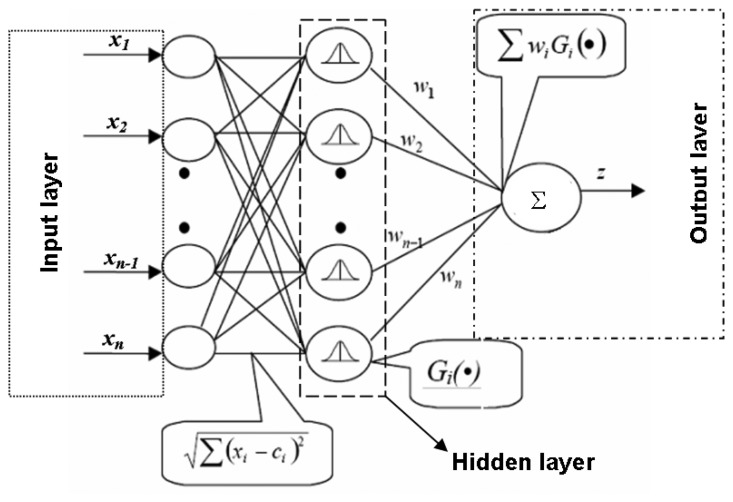
RBF neural network scheme.

A conventional RBF architecture has been used due to its simplicity. Figure 10 shows the structure of this kind of neural network. This neural architecture consists of a non-linear hidden layer and a linear output one, in which the contribution of the signals pondered by the *G_i_* Gaussian activation functions of the neurons are combined to provide the *S_11_* parameter at the working frequency. These functions are defined by the ***c****_i_* adaptive centroids and a constant variance value for all the Gaussians.

The ***c****_i_* centroids determine the segmentation of the input space of the ***x*** input vector. The components *x_i_* of vector ***x*** are fed as inputs to the neural network. This input is processed by the Gaussians to give *G_i_*, the output of the hidden level. The output of the network is obtained by means of the vectorial expression **W· G** in the output linear level, ***W*** being the weights of the linear layer. In this case, the input vector is formed by the eight voltage values provided by the LTC5530 detectors, whereas the output of the network estimates |*S_11_|*. A similar scheme is used for the estimation of *S_11_* phase.

RBFs are supervised neural networks whose structure provides a solution to the local interpolation of non-linear functions [15]. This is the case of the generic function *S_11_*(***x***), ***x***={*x_1_*,.., *x_8_*}, considered in this work –see Figure 14– since it does not have a linear behaviour. Although the activation of the neurons in the RBF model is carried out by radial basis functions, this model has a linear expression for the estimation of *S_11_*. Therefore, for each input vector ***x****(k)*, the estimation of *S_11_* is given by equations (2) and (3).

(2) $$s_{11}(k)=\overset{M}{\underset{j=1}{\text{∑}}}w_{j}G_{j}(k)$$

(3) $$G_{j}(k)=\exp\left(\frac{\sqrt{\underset{i}{\text{∑}}{\left(x_{i}(k)-c_{j}^{i}\right)}^{2}}}{\sigma_{j}^{2}}\right)$$

where *G_j_*(*k*) is the output of the *j^th^* Gaussian radial function at input ***x****(k)*, *c_j_* and *σ_j_* are the centre and standard deviation of *G_j_*(*k*), *w_j_* is the weight value associated to *G_j_*, *k* is the training example and *M* the number of neurons of the network.

The estimation of *w_j_* is carried out by using the gradient descent algorithm to minimize the cost function described in Equation (4).

(4) $$H=\overset{N}{\underset{k=0}{\text{∑}}}{\left(S_{11}^{T}(k)-S_{11}^{M}(k)\right)}^{2}$$

where *S^T^_11_(k)* and *S^M^_11_(k)* represent the theoretical and measured values for the parameter *S_11_*, for both magnitude and phase. *N* is the number of examples used for training the neural network.

As a conclusion, the application of the RBF neural network to the estimation of *S_11_* permits to obtain, after the training stage, the optimal values for *w_j_*. In the operation stage, equation (2) supplies the approximation of *S_11_(k)* from DC voltages provided by power sensors.

## 3. Experimental set-up

**Figure 11.**
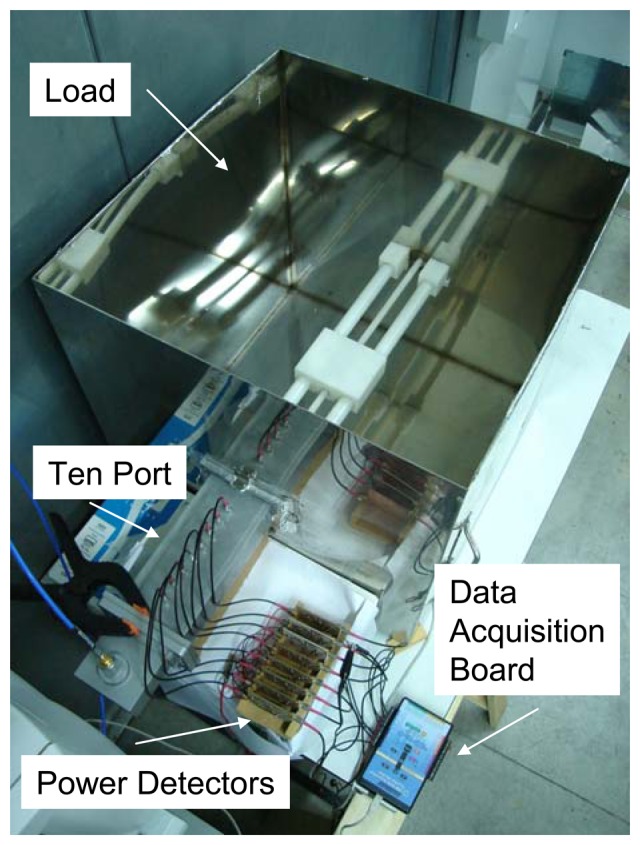
Experimental set up for both sensor calibration and validation.

Figure 11 shows the experimental set up implemented for the ten-port calibration and validation. A multimode 60 × 60 × 60 cm^3^ cavity has been employed for testing the sensor under low power conditions, and to provide different *S_11_* values for calibration purposes. Two DC supply sources were employed for biasing the *LTC5530* power sensors.

A power level of 0.5 watts was introduced by the ZVRE Rohde & Schwarz VNA at 2.45 GHz across Port 1. The neural network was implemented in a personal computer running Matlab™ Neural Network Toolbox™ routines. The personal computer was connected to the VNA by a USB GPIB communication board, and to the data acquisition board through conventional USB connectors. The error for RBF training was obtained by comparing the reference *S_11_* value provided by the VNA and the value computed by the neural network as shown in Equation (2). This error was minimized when optimal *w_j_* values were found after the optimization process.

**Figure 12.**
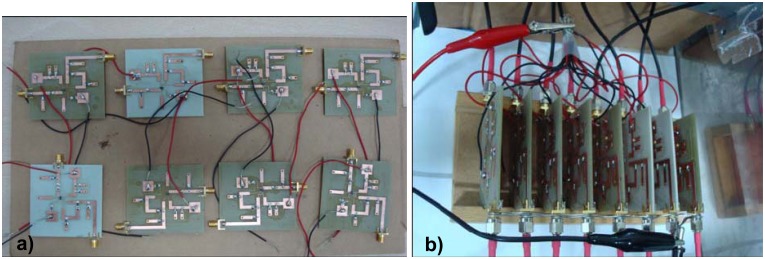
a) Manufactured power sensor boards. b) Rack disposition with biasing and ground connections.

**Figure 13.**
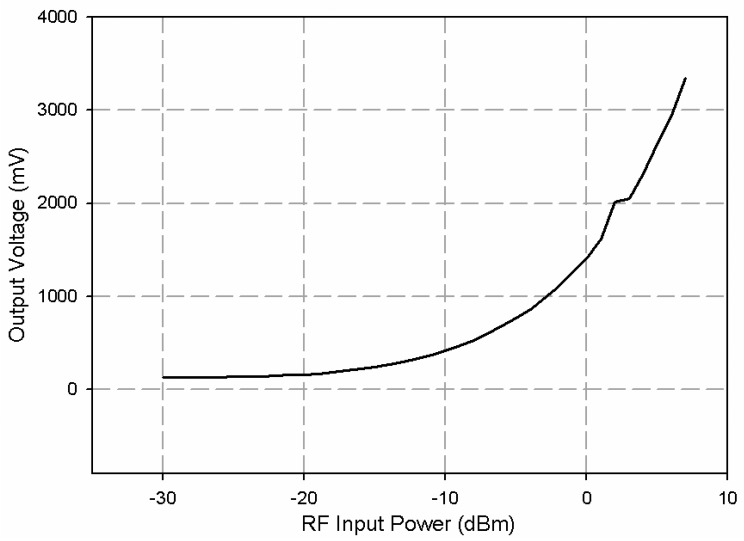
Measured RF power-DC voltage conversion curve at 2.45 GHz.

Figure 12 shows the *LTC5530* circuit boards and common ground and biasing connections. The power-DC voltage conversion curve was measured by using the VNA as an RF signal generator, and a conventional multimeter for measuring the output voltage. This was needed since conversion curves were provided at 2 GHz but not at 2.45 GHz. Figure 13 shows the experimental power voltage conversion curve at 2.45 GHz. This verified the correct implementation of power sensor boards.

Coaxial probes were designed to obtain a maximum input power level at the power sensor, equal to -10 dBm. The coupling of waveguide transmitted power in the coaxial probes was adjusted by changing the length of these coaxial probes within the waveguide. In this case, the coaxial probe coupling was adjusted to -17 dBm with a coaxial probe length equal to 16 mm.

During the calibration process, the RBF neural network was trained with *N*=255 training patterns. These patterns were formed by an input matrix with *N x 8* elements corresponding to the DC voltages from the power sensors and an output matrix with dimensions *N x 2*, corresponding to the magnitude and phase of reference value of *S_11_* provided by the VNA. A variable load provided different values for *S_11_* The phase and magnitude of training data for the *S_11_* covered the ranges [0, 2π] and [0, 1], respectively. The number of Gaussian neurons was fixed to 60. This number was selected after some trials, because it gave an adequate balance between learning and generalization. The centers of the Gaussian neurons were chosen to be equally spaced, covering the whole range of the inputs. For all the neurons, *σ_j_* was set to 0.3466. That is equivalent to say that the spread factor of the Gaussians is of 0.1, inside of the hypercube of the normalized inputs.

## 4. Results and Discussion

**Figure 14.**
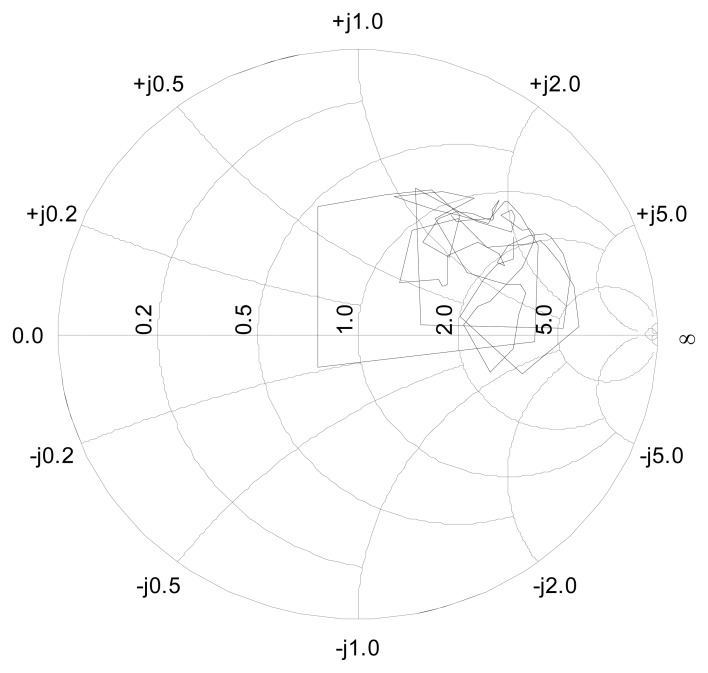
Smith chart representation of *S_11_* complex data used in the experimental training stage.

Figure 14 shows the training data for the *S_11_* parameter provided by the VNA. These reference data are represented in the so called Smith chart that shows *S_11_* both in magnitude and phase. Once the network has been trained, the weights of the RBF neural network are optimized and the sensor is ready to estimate the value of *S_11_* only from the output values of the power sensors.

**Figure 15.**
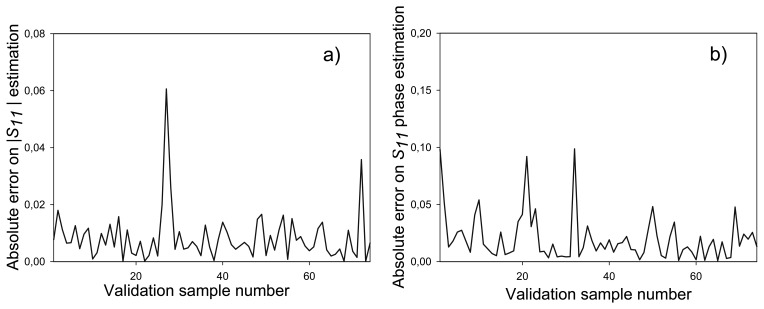
Validation error for 74 different validation patterns. (a) *S_11_* magnitude. (b) *S_11_* phase.

In this case, 74 patterns that had not been used during the training process have been employed for validation purposes. The *S_11_* estimated values provided by the ten-port reflectometer have been compared to the ones measured by the VNA, both in magnitude and phase. Figure 15 shows the absolute error obtained for this comparison, resulting in average absolute error of 6.2 × 10^-3^ for the magnitude and of 2.3×10^-2^ for the phase of *S_11_*. As it can be observed in Figure 15, this error is very small and therefore very accurate measurements can be made with the proposed reflectometer. The phase error is bigger than the magnitude one, since phase can vary from 0 to 2π radians whereas magnitude variation ranges from 0 to 1.

**Figure 16.**
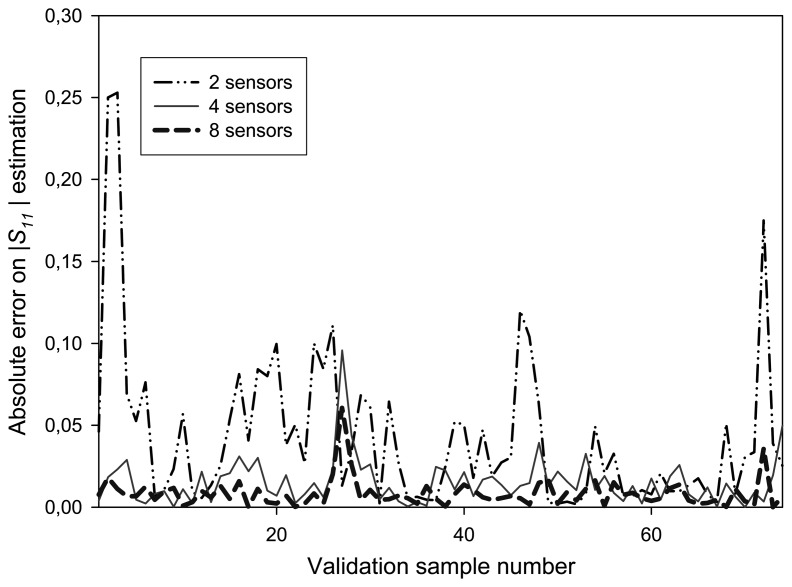
Behaviour of the reflectometer when different numbers of detectors are considered.

In order to evaluate the behaviour of the ten-port when some detectors are unable to measure because of electronics faults, several tests have been carried out by using only a reduced number of diodes as valid sensors to measure magnitude and phase for *S_11_*. The output of the faulty detectors is defined as a constant value for all the training positions. The defective sensors were chosen in a random way. Some of the results obtained for the absolute error of |*S_11_|* when compared to VNA measurements are represented in Figure 16, where 255 training patterns and 74 validation measurements have been considered.

From the curves in Figure 16, it can be observed that the ten-port structure is able to correctly predict the *S_11_* function even when some detectors do not work properly, provided that the working detectors allow an appropriate sampling of the stationary wave, as stated by the Shannon theorem [16]. It is noticeable that the error observed when all the power sensors are working is lower than those obtained with 4 power sensors, which is the configuration of a conventional SPR. However, the error observed when only two power detectors are in use is not acceptable and a minimum of 4 power detectors should be used when carrying out the measurements. This is an expected result, because at least four samples along its wavelength are needed to correctly sample a wave without losing information. Therefore, these results show that it is possible to reconfigure the sensor software structure by training again the RBF neural network in order to correctly operate even when some of the power sensors are broken or faulty.

## 5. Sensor application to microwave oven optimization

**Figure 17.**
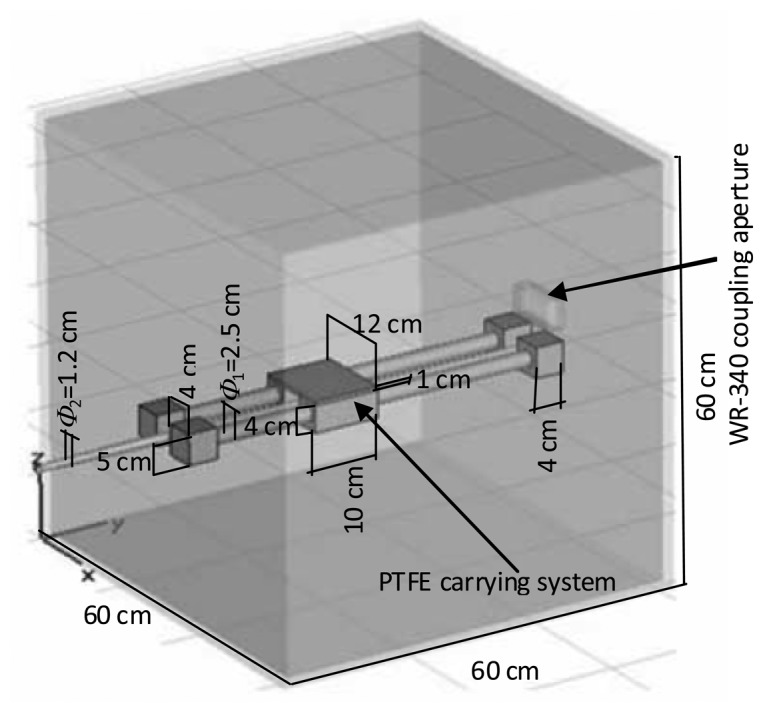
Scheme of the multimode microwave oven employed during optimization process.

Finally, as an application of the sensor capacities, the ten-port reflectometer has been used in order to optimize the behaviour of a 60 × 60 × 60 cm^3^ microwave oven. The sensor was used to monitor the value of the *S_11_* magnitude in real time for different positions of a 250 cm^3^ cylindrical water sample. Figure 17 shows the scheme of the employed cavity. As it can be observed, the optimization process is focused only on one dimension along the main axis of the oven. It gives a partial solution to the problem for the considered oven because the optimization process is completed only when the three dimensions are considered for the algorithm. Although the symmetry of the process permits to consider the *y*-axis as the dimension in which the maximum variations for the electromagnetic field are produced, a more accurate result can be found for experimental platforms with a cartload displacing along the 3 axis inside the oven.

**Figure 18.**
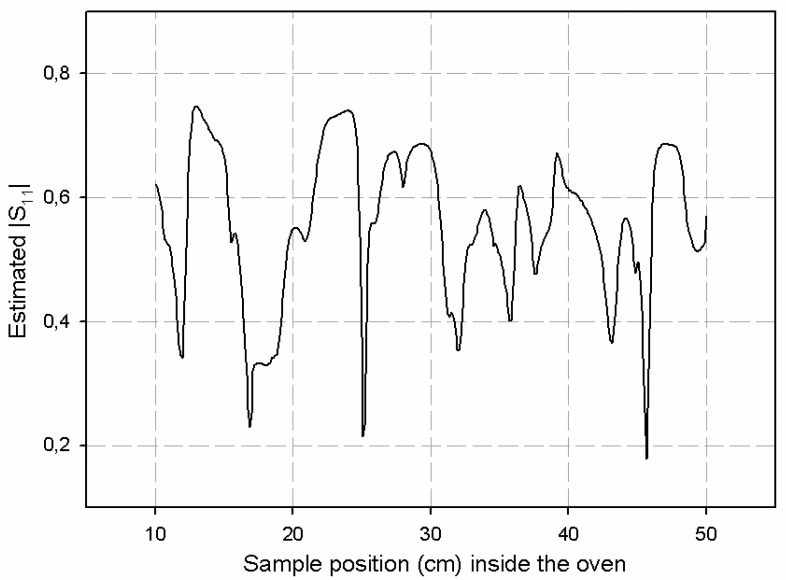
|S_11_| response versus sample position within the multimode oven.

Optimization was carried out by properly placing the sample at the optimum position along the PTFE carrying system. Figure 18 shows the results obtained by the sensor for the *S_11_* magnitude when 0.5 watts were employed as microwave power source and the sample was moved with the PTFE carrying system. The movement was carried out by placing the sample as near as possible to the WR-340 coupling aperture and then moving the sample away from that position. As it can be observed in Figure 18, values lower than 0.2 can be obtained for *S_11_* magnitude for sample distances around 46 cm away from the coupling aperture. On the contrary, other sample positions may lead to reflection coefficient values up to 0.75. Therefore, the employment of this ten-port reflectometer allows, once it is calibrated, the monitoring of the reflection coefficient of microwave ovens in real time. It implies to improve the energy use and to protect the microwave sources from undesired reflections that may damage them.

## 6. Conclusions

In this work the simulation and validation of a ten-port prototype microwave sensor has been presented. This low-cost sensor allows the reflection coefficient estimation in real time and has been used in order to optimize microwave multimode ovens. The ten-port has been implemented in waveguide technology, which is the usual transmission medium used in power applications. A RBF neural network has been used to learn the relationship of power sensor output signals and the reference values of the reflection coefficient.

Tests have shown that a very good estimation of the reflection coefficient can be obtained both for magnitude and phase, with accuracy comparable to high-cost laboratory equipment such as VNAs. Although results are shown for low power levels, the procedure can be readily extended to higher power levels by changing the coupling level of coaxial probes within the waveguide. An advantage shown by the sensor versus conventional waveguide reflectometers is that it can be readjusted even if some of the power detectors fail, still providing precise results. Additionally, linearization processes or theoretical modeling of the device are no longer needed for calibration, since neural networks have the ability to learn and correct such problems.
